# Systems Genomics of Thigh Adipose Tissue From Asian Indian Type-2 Diabetics Revealed Distinct Protein Interaction Hubs

**DOI:** 10.3389/fgene.2018.00679

**Published:** 2019-01-08

**Authors:** Pradeep Tiwari, Aditya Saxena, Nidhi Gupta, Krishna Mohan Medicherla, Prashanth Suravajhala, Sandeep Kumar Mathur

**Affiliations:** ^1^Department of Biotechnology and Bioinformatics, Birla Institute of Scientific Research, Jaipur, India; ^2^Department of Endocrinology, Sawai Man Singh Medical College and Hospital, Jaipur, India; ^3^Department of Chemistry, School of Basic Sciences, Manipal University Jaipur, Jaipur, India; ^4^Department of Biotechnology, Institute of Applied Sciences and Humanities, GLA University, Mathura, India; ^5^Department of Biotechnology, The IIS University, Jaipur, India

**Keywords:** systems genomics, type-2 diabetes, adipocyte tissues, phenotypic traits, interaction networks

## Abstract

We performed a systematic analysis of genes implicated in thigh subcutaneous adipose tissue of Asian Indian Type 2 Diabetes Mellitus (AIT2DM) and created a phenome-interactome network. This analysis was performed on 60 subjects specific to limb thigh fat by integrating phenotypic traits and similarity scores associated with AIT2DM. Using a phenotypic attribute, a contextual neighbor was identified across all the traits, *viz*. body mass index (BMI) statistics, adipocyte size, lipid parameters, homeostatic model assessment- insulin resistance (HOMA-IR), HOMA-ß. In this work, we have attempted to characterize transcription signatures using the phenome-interactome maps where each of the traits under study including the intermediary phenotypes has a distinct set of genes forming the hubs. Furthermore, we have identified various clinical, biochemical, and radiological parameters which show significant correlation with distinct hubs. We observed a number of novel pathways and genes including those that are non-coding RNAs implicated in AIT2DM.We showed that they appear to be associated with pathways, viz. tyrosine kinase JAK2, NOTCH thereby recruiting signaling molecules such as STAT5 and Src family kinases on the cell surface regulated them and our analyses comprising significant hubs suggest that thigh subcutaneous adipose tissue plays a role in pathophysiology of AIT2DM.

## Introduction

Asian Indian Type 2 diabetes mellitus (AIT2DM) and its association with obesity is relatively well-known showing a distinct disease phenotype. With over 415 million people diabetic worldwide as of 2015, it is predicted that diabetic population in India is alarming and would rise to 140 million by 2040 (Whiting et al., 2011; Joshi, 2015). Higher incidence of diabetes is reported in India as compared to western countries with several studies showing that Indians are associated with lower body mass index (BMI) compared to their western counterparts (Misra et al., 2015). This scenario might make even relatively lean Asian Indian adults with a lower BMI at equal risk as those who are obese. While studies performed on Indian subjects have shown characteristic clinical phenotypes ranging from BMI statistics, adipocyte size, lipid parameters, homeostatic model assessment- insulin resistance (HOMA-IR), HOMA-ß etc., they have a different body composition compared to other racial groups and show typical lean-fat phenotype (Jain et al., 2013; Unnikrishnan et al., 2014). This phenotype is characterized by more abdominal fat deposition and lesser peripheral fat. Though the AIT2DM subjects show high insulin resistance, hyperglycemia, and dyslipidemia, it is expected that the aforementioned characteristic phenotypic traits are scattered all through the body fat distribution. While diabetics in AIT2DM population are known to typically possess less limb fat when compared to non-diabetics, their diabetic risk is generally attributed to higher visceral fat content. Recent evidences have raised questions on the role of omentum fat in insulin resistance and diabetes. For example., it is the ectopic liver fat which showed association with insulin resistance and even surgical removal of omental (visceral) fat had no effect on insulin resistance (Sattar and Gill, 2014). In addition, visceral adipose tissue is a significant risk factor for obesity-related metabolic disorders and hence its reduction would serve as a key goal for obesity reduction (Fabbrini et al., 2009). The nutrient overflow hypothesis of Asian Indian phenotype further suggests that it is the inability of protective adipose tissue depots like thigh to contain excess calories which leads to ectopic fat deposition in liver and muscle contributing to dysmetabolic states (Sniderman et al., 2007). Therefore, a detailed study employing transcriptomic profiling of thigh adipose tissue was needed to understand this complex Asian Indian phenotype.

In the recent past, studies on transcriptional profiles using microarray and RNA-Seq have provided candidate differentially expressed genes (DEG) (O'Brien et al., 2012). However, the association of AIT2DM with intermediary phenotypic traits from genome wide association studies (GWAS) is limited (Lee and Lee, 2014; Fuchsberger et al., 2016; Srivastava et al., 2016; Wahl et al., 2017). We have earlier developed a systems genomic approach correlating genome to phenomes underlying AIT2DM pathophysiology and identified the overrepresented genes associated with AIT2DM (Jain et al., 2013). With the advent of next generation sequencing, the genetic variation underlying the intermediary phenotypic traits associated with diabetes is beginning to be understood (Scott et al., 2016; Shungin et al., 2017). We hypothesize that a module of co-expressed genes related to thigh subcutaneous adipose tissue dysfunction with significantly enriched genes (coding and non-coding) and their interactions would be useful to understand the molecular qualitative factors associated with the AIT2DM (Geng and Tan, 2016). This regulatory role would be crucial when the non-coding RNAs are known to be interacting with expressed transcripts which can throw insights on their role related to various functions (Suravajhala et al., 2015; Xu et al., 2017). While we attempted to make a phenome-interactome network considering thigh subcutaneous adipose tissue of AIT2DM, this is perhaps the first such study for Asian Indian population.

## Materials and Methods

### Study Subjects

Transcription profiling for 60 samples, *viz*. 30 controls and 30 diabetic subjects was performed to study the expression level of genes from the thigh subcutaneous adipose tissues (GEO Accession # GSE78721). The inclusion and exclusion criteria on control and case subjects with variables have been defined appropriately (Table 1). In total, 30 control and 30 diabetic subjects undergoing femur bone surgery for traumatic fracture were recruited (Supplementary Table 1). Approval from institutional ethics committee and informed written consent before participation of subjects was obtained. This study protocol was approved by the ethical committee of SMS hospital, Jaipur and the Indian Council of Medical Research (ICMR), New Delhi. All methods were performed in accordance with the relevant guidelines and regulations.

**Table 1:** Inclusion and exclusion criteria for the study subjects.

**Condition**	**Inclusion criteria**	**Exclusion criteria**
Diabetic subjects	Non obese (BMI < 30) type-2 diabetics diagnosed as per American Diabetes Association (American Diabetes Association, 2012) criteria undergoing femur bone surgery.	Presence of infection, malignancy, and drugs affecting body fat/insulin resistance or adipokine expression like glitazones, metformin and glucocorticoids.
Non-diabetic	Age, sex, and BMI matched normal glucose tolerance subjects undergoing femur bone surgery.	Presence of infection, malignancy, and drugs affecting body fat/insulin resistance or adipocytokines expression like glitazones, metformin and glucocorticoids.

### Clinical, Biochemical, Radiological Assessment, and Sample Collection

The anthropometric measurements such as body weight, height, waist to hip (W: H) ratio, and BMI were obtained by standard methods performed on all samples. Supine blood pressure was measured using mercury sphygmomanometer after 10 min of rest. Blood samples were obtained at 8:00 am after an overnight fast of at least 8 h. The adipose tissue biopsy was taken from either the left or the right thigh depending on the site of the fracture, the primary indication of the surgery. Various biochemical parameters (such as serum glucose, lipid profile, triglycerides, LDL, HDL and VLDL) were measured on Kopran AU/400 fully automated analyzer (Supplementary Table 1). Serum insulin was measured using chemiluminescent immunometric assay (Immulite 2000 machine, with kits supplied by the manufacturer). HbA1c was measured by turbimetry method using BioSystems kits. HOMA-IR and HOMA-ß were taken as the parameter of insulin resistance and secretion respectively and were estimated using standard formulas respectively. Non-esterified fatty acids (NEFA, Rendox Laboratories Ltd UK kits) were measured by the biochemical method. High sensitivity C reactive protein (hsCRP, Diagnostics Biocheme Canada Inc., Canada), Leptin (Lab systems Diagnostic Oy, Finland), Adiponectin (Diagnostics Biocheme Canada Inc., Canada), were estimated by ELISA kits. Body fat content was assessed by Dual energy X-ray absorptiometry (DXA) using Hologic Explorer model (S/N91395 make).

### Microarray and Transcription Profiling

The microarray analysis was performed on raw data with a focus to prioritize DEGs. The amplified RNA was fragmented and hybridized to Affymetrix GeneChip PrimeView Human Gene Expression array containing 49395 probes, representing all known transcripts of the human genome. The raw data generated was quality checked (QC) using Affymetrix expression console and Transcriptomic Analysis Suite (TAS) 4.0 software. Post scanning the hybridized arrays and resulting biological replicates for the two sets of case-control groups, bioinformatics analysis using R and Bioconductor was done. The raw data was first normalized using robust multiarray analysis (RMA) and two fold change cut-off was used to infer the differentially up-regulated or down-regulated genes using the pipeline (Supplementary Table 4). Furthermore, we performed a non-specific filtering step in which we calculated overall variability across array of each probe set, regardless of the sample label. For this we used the function *rowSds* from package *genefilter*, which calculated the standard deviation for each probe followed by the function *shorth* from the same package which yielded the shortest interval containing half of the data that can be considered a reasonable estimator of the “peak” of a distribution. We discarded those probe sets whose standard deviation was below 0.54. Further, all probes without Affymetrix id, Entrez id or GO-term were not considered as these could not be utilized in downstream analysis. Necessary sub-setting was carried out for DEG analyses with modified t-statistics based on empirical Bayes moderation approach implemented in the *eBayes* function using *limma* package. Genes with *P*-value threshold < 0.05 were considered differentially expressed. For further downstream analyses, we converted the temporary probe ids that we generated from RMA to affymetrix probe ids and further proceeded for Gene Ontology (GOEAST) and Kyoto Encyclopedia of Genes and Genome (KEGG) analyses. A statistical significance (probability) of 0.05 was obtained for analyzing the data all through.

### Real Time PCR

For the candidate DEGs, the reliability of microarray analyses was tested by Reverse Transcriptase- PCR (RT-PCR) on selected genes (Supplementary Table 5). Oligos (primers) were raised using universal probe library from Roche and synthesized as per appropriate melting temperature (Tm), GC%, synthesized by Imperial Life Sciences, Gurugram, India. The primers synthesized were double checked to get amplimer product. RNA was isolated from the adipose tissue using Qiagen RNeasy Mini Kit (Cat No. 74104). The quantitation of samples was performed using microfluidic based capillary electrophoresis system (BioRad Experion). cDNA Synthesis was done using QuantiNova Reverse Transcription Kit (Cat No. 205411) and real time PCR was done using QuantiNova Probe PCR Kit (Cat. No 208252).

### Systems Genomics Integration

We used contextual hub analysis tool (CHAT) (Muetze et al., 2016) and integrated Complex Traits Network (iCTNet) (Wang et al., 2011) along with vizmap parameters, plug-ins from Cytoscape to understand the association of DEGs with phenotypic traits. In order to identify transcriptionally regulated genes, we considered the genes enriched from our list as a query against cytoscape. The contextual information of the neighboring interacting partners was checked for in agreement with the *p*-value (Bonforreni < 0.05). The pre-processing involved reducing the noise resulting from CHAT variations and subsequently determining the actual biological change or flux of DEGs forming hubs. Identification of DEG hubs with changes in expression profiles in diseased/undiseased conditions was performed keeping in view of the topological/contextual neighbors and a QQ plot was generated. The list of DEGs forming significant hubs against the inferred *p*-value led to the identification of crucial genes. Furthermore, cluster analysis for co-expressed genes forming the hubs based on the phenotypic traits, *viz*. HOMA-IR, HOMA-B, Leptin, ß-cell dysfunction were considered using iCTNet (Wang et al., 2011) and the contextual hubs which share similar expression/biological features were clustered together. This integration of disparate large-scale data generated in the form of DEGs was inferred with phenotypic traits.

## Results and Discussions

In this study, a detailed analysis of DEGs, phenotypic traits and their role in diverse pathways related to thigh fat has been extensively carried out. From the clinical, biochemical, and anthropometric assessment, there was no significant difference between diabetics and controls groups with respect to gender, age, and weight. However, diabetics had relatively higher BMI, waist circumference, plasma glucose, insulin, and HOMA-IR levels and there was no significant difference in levels of adipo-cytokines between diabetics and controls (Supplementary Table 1). The gene expression analysis showed a significant number of enriched genes which were categorized to pathways based on the phenotypic traits (Supplementary Table 2). The enriched genes in the form of DEGs were classified between the three distinct diabetic metabolic disorders (Obesity, Type-1 Diabetes, and Type-2 Diabetes) which we detailed in the later sections. However, we have considered the top enriched genes in the form of LINC01128, SAMD11, KLHL17, PLEKHN1, ISG15, AGRN, MIR200B, MIR200A, MIR429, TTLL10, B3GALT6, SCNN1D, PUSL1, CPTP, TAS1R3, VWA1, SSU72 ,MIB2, CALML6, and GABRD. A select 10 genes including two of them being the housekeeping genes were quantitatively confirmed using RT-PCR.

**Figure 1:**
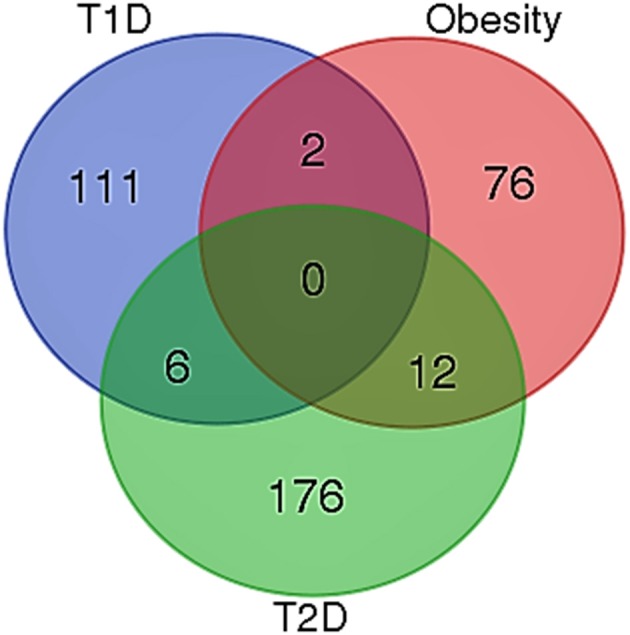
Venn diagram showing the number of genes across the three contexts. Two genes, viz. POMC and ITPR3 have been enriched between T1D and Obesity; six genes, *viz*. HNF1A, RASGRP1, DLK1, CENPW, GLIS3, and SH2B3 between T1D and T2D; and 12 genes, *viz*. PROX1, MC4R, ENPP1, TMEM18, FTO, FAM150B, IRS1, PPARG, SPRY2, TCF4, LYPLAL1, and NYAP2 between obesity and T2D.

**Figure 2:**
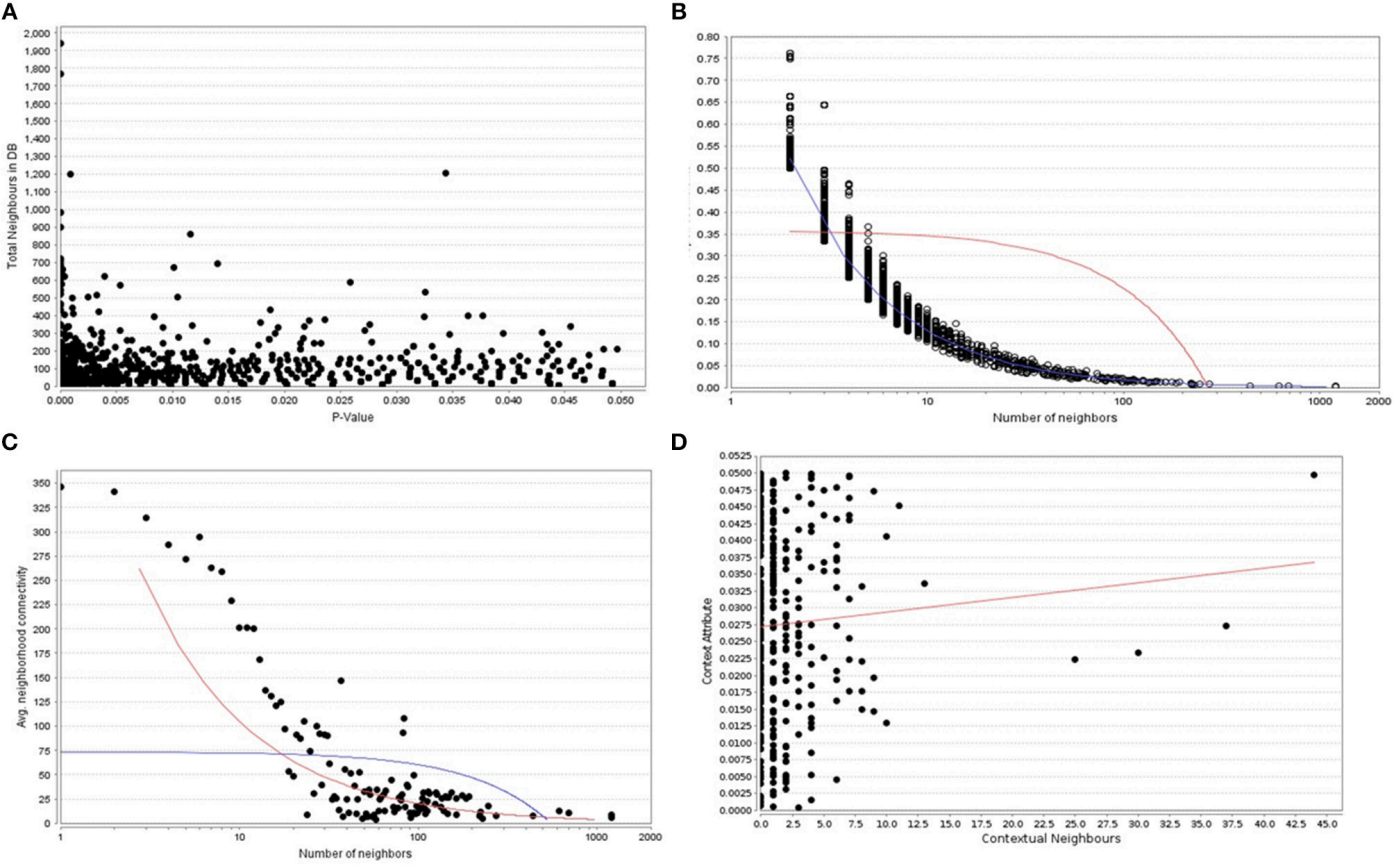
**(A)** The P -values plotted against the total neighboring DEGs, **(B)** The number of neighbors mapped to the total coefficients, **(C)** The number of distinct neighbors against the average neighborhood connectivity from DEGs, and **(D)** contextual neighbors plotted against the contextual attribute.

When the significantly enriched genes from RMA (Supplementary Table 3a) were provided as an input for the CHAT analysis, we obtained 358 genes with a contextual attribute (bonferroni < 0.05). When this dataset was facilitated with “diabetes” as a search ontology term in iCTNet, it further produced 416 nodes and 497 edges with an average of 2.3 neighbors per node (Supplementary Table 3b). Among the interaction pairs, we observed 199 high confident pairs related to type 1 and type 2 diabetes (Supplementary Table 3c). On further analysis from these three contexts, two genes, *viz*. POMC and ITPR3 have been enriched between T1D and Obesity; six genes, viz. HNF1A, RASGRP1, DLK1, CENPW, GLIS3, and SH2B3 between T1D and T2D; and 12 genes, viz.PROX1, MC4R, ENPP1, TMEM18, FTO, FAM150B, IRS1, PPARG, SPRY2, TCF4, LYPLAL1, and NYAP2 between obesity and T2D indicating that the genes enriched between these contexts are highly significant (Supplementary Table 3d; Figure 1). Notably, RAS Guanyl Nucleotide-releasing Protein or RASGRP1 enriched in both T1D and T2D is a member of a family of genes characterized by the presence of a Ras superfamily guanine nucleotide exchange factor (GEF) domain activating the Erk/MAP kinase cascade and regulating T-cells and B-cells development besides being associated with high fat induced obesity and a causal of susceptibility to systemic lupus erythematosus (SLE) (Jung et al., 2015) (Supplementary Table 3e). On the other hand, the well-known peroxisome proliferator-activated receptor -γ (PPAR-γ, a.k.a PPARG) is the most significantly enriched genes between T2D and obesity contexts is a regulator of adipocyte differentiation and has been implicated in the pathology of numerous diseases including obesity, diabetes, atherosclerosis and cancer (Larsen et al., 2003). Whereas, certain genes responsible for adiposity and lipid catabolism were down-regulated, contextual set of genes based on the number of edges yielded their expression profiles with top KEGG pathways. The *P*-values against the total neighbors in CHAT analyses show that the density of neighbors decrease with increase in adjusted *p*-value and the DEGs mapped with the *P*-value coefficient (*P* < 0.05) are in agreement with each other (Figure 2). From the contextual analysis, we identified four significant genes (with adjusted *P* < 0.05), *viz*. OBSL1, an Obscurin which is associated with insulin-like growth factor binding protein (IGFBP2), ZC3H15 (HT010), a zinc finger CCCH-type containing 15, PHLL1, a photolyase like protein and hnRPR, heterogeneous nuclear ribonuclear protein. Whereas, OBSL1 expression is directly associated with insulin growth factors, the latter three proteins are either associated with circadian rythms, abdominal aortic aneurysms (AAA) leading to negative side of diabetes (Jones et al., 2017) or stimulation of transcription factors associated with diabetes (Lo et al., 2015). In addition, research on transcription factor biology associated with genetic pathways especially in the etiology of diabetes has seen major breakthroughs in the recent past (Dai et al., 2017; Yang et al., 2017). However, the transcriptional signatures associated with the interacting genes could be measured first based on a phenome interactome network. This transcriptomic data combined with the phenome-interactome map would facilitate identifying the prognosis underlying diabetic trait complications.

### Highly Connected Nodes Relevant to Phenotypic Traits

**Figure 3:**
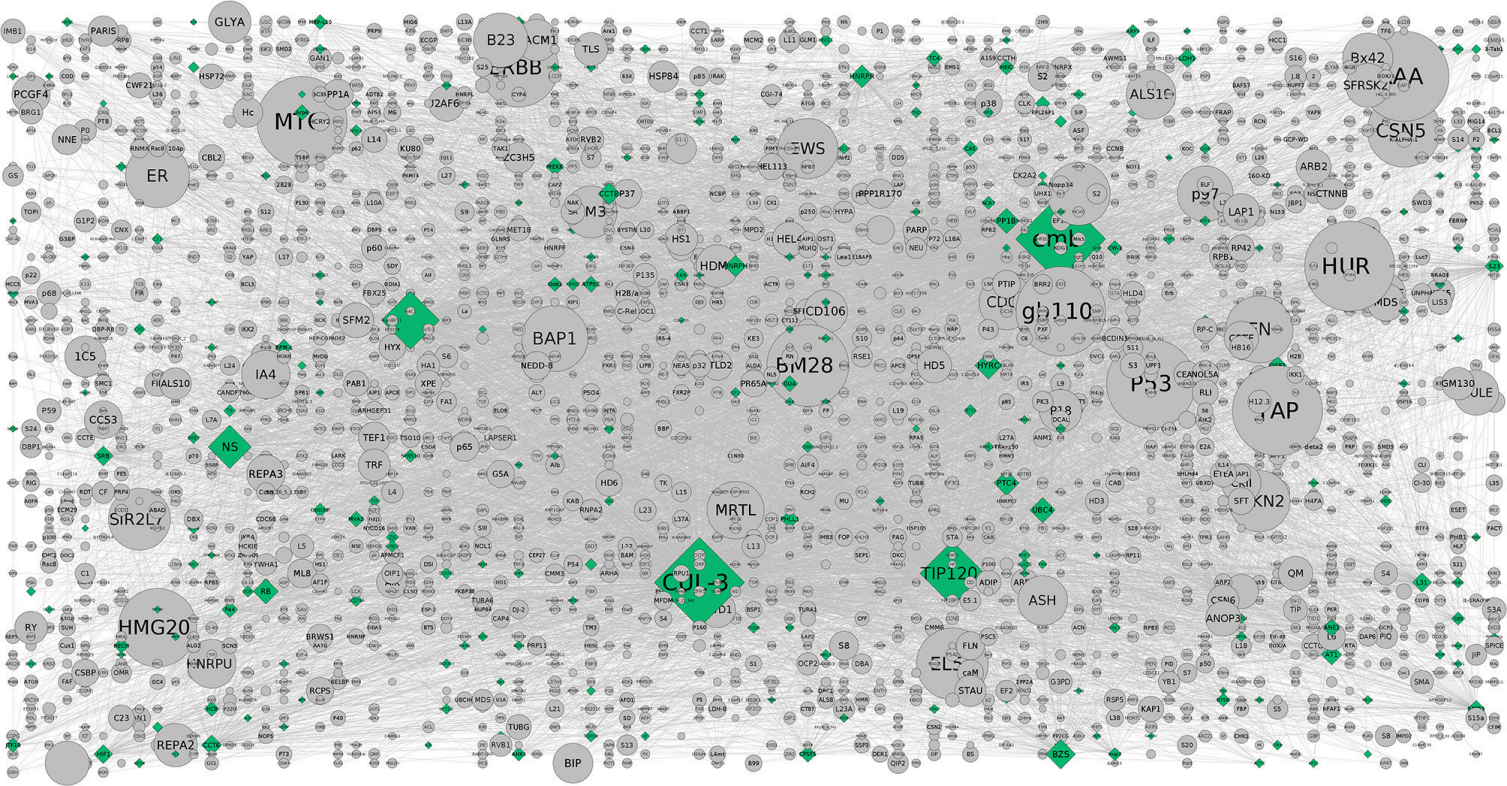
The CHAT generated grid network with DEGs shown in (contextual) green and (non-contextual) gray colors. The contextual nodes are in pyramid shape while the non-contextual nodes are circled with the size indicating the extent of contextuality. For example, CUL-3 and TIP120 are contextually important genes relevant to the pathways and are contextually important with a good clustering co-efficient and *P*-value (< 0.05) (Supplementary Table 3b).

**Figure 4:**
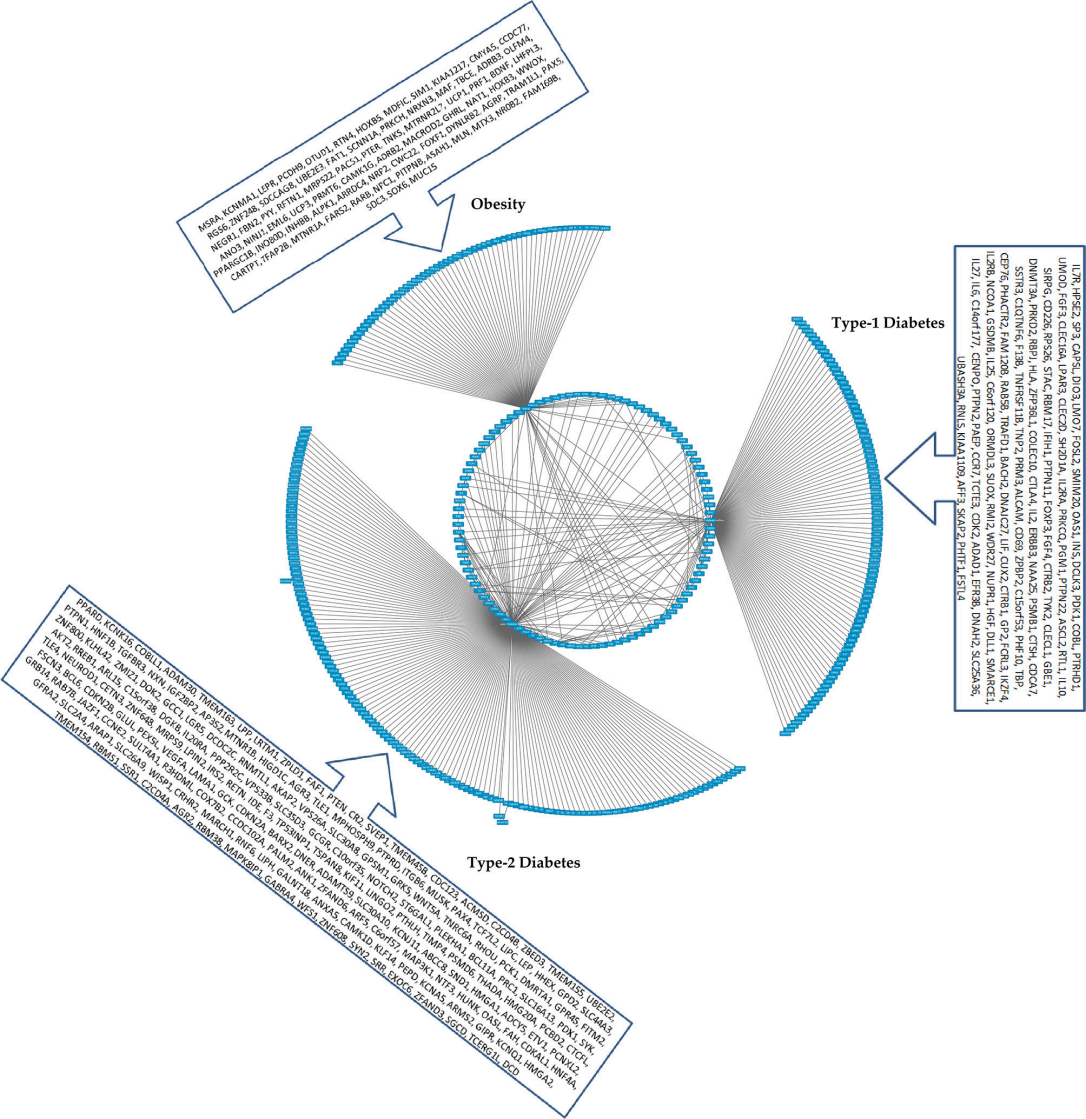
The CHAT generated bundled circular layout with phenotypic associations and the DEGs. For list of DEGs inherent to the pathways and the contexts (nodes and connecting edges), please see Supplementary Tables 2, 3. The contextual parameters are shown in a horizontal list of nodes below the interaction network.

From CHAT networks, we observed a significant number of hubs that are topologically important and preferentially associated with a range of phenotypes of interest. When CHAT was used to compare contextual and degree-based hubs, the latter seem to be enriched in pathways related to obesity and adipogenesis due to the fact that the proteins tend to be more connected to each other when compared to random. In comparison, contextual networks are considerably observed to be more “biologically distinct” as they are highly connected than a connection specific to context of interest. Furthermore, centrality/betweenness analysis of the co-expression networks and pathways were validated with the contextual networks and compared with the attributes (phenotypic traits). An average of 2.5 connected nodes per attribute was seen showing a preferential association with a range of phenotypes of interest (Figures 3, 4). However, the relative importance of a hub node could change depending on the biological context further suggesting the diverse pathways were associated with them. This analysis showed that such contextual hubs were considerably more biologically relevant than degree-based hubs which tend to be biased toward nodes that are highly connected in general rather than in the specific context of interest. The co-expression network containing DEGs between AIT2DM subjects and normal controls consisted of sizeable number of nodes and edges with hubs further evaluated by assessing the co-expression networks. We observed hub genes associated with AIT2DM, which may be considered as potential biomarkers for early detection and therapy for adiposopathy or “sick thigh fat” mediated diabetes (Supplementary Table 2). For example, comparing the existing DEGs with already published GWAS of Indian population (Tabassum et al., 2013), MAP3K1 and ADAM12 were observed to be enriched in terms of contextual importance and their association was found to be statistically significant. These contextually important DEGs were not identified by previous GWAS; however some of the interacting partners in the form of DEGs (example ADAM12) were associated with similar effect from previous reports (*P*-value of < 0.05).

### Identifying Pathways for the Causality

**Figure 5:**
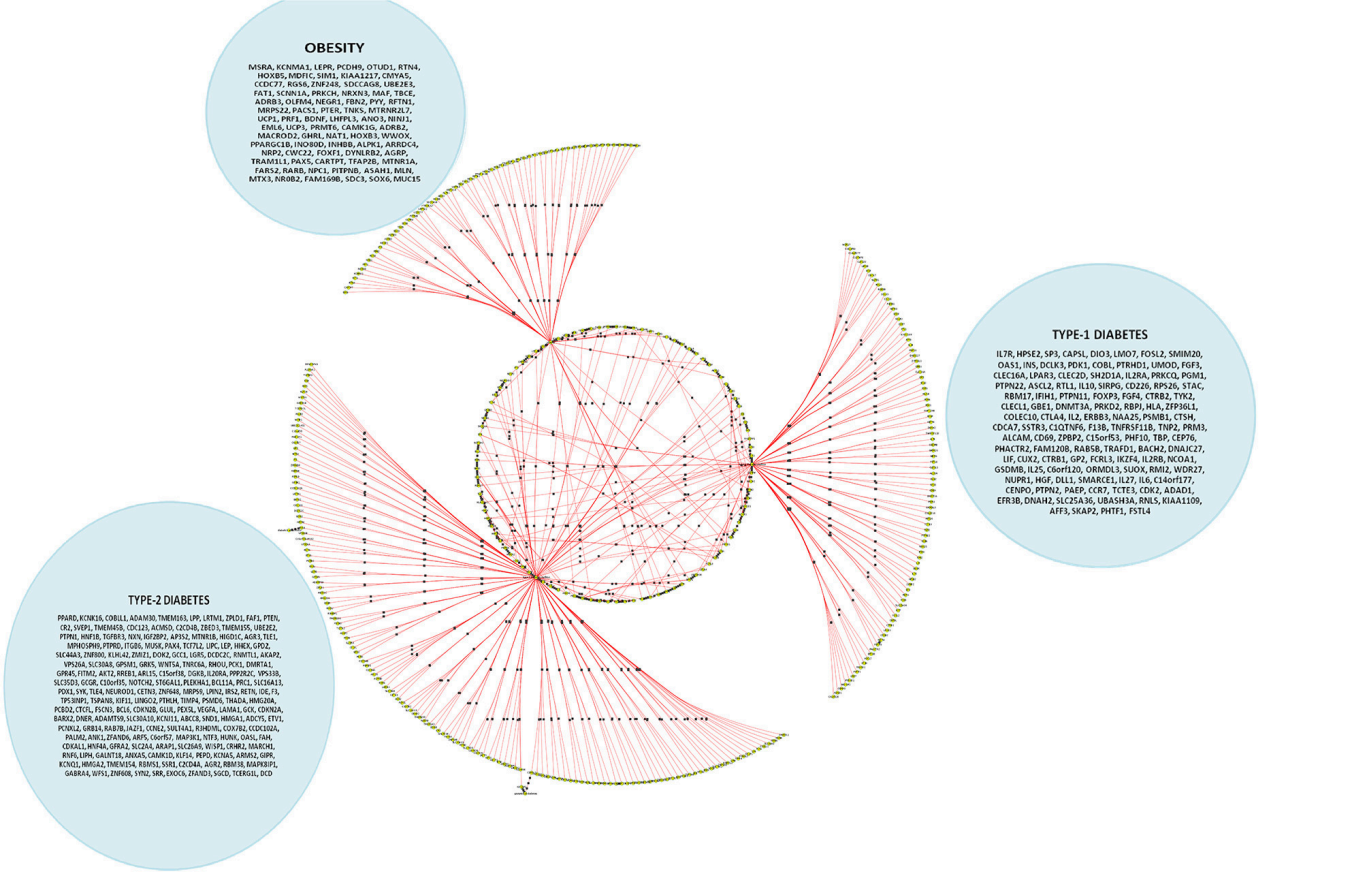
A cohort of genes bundled across three contexts, *viz*. type 1 diabetes mellitus (T1D), type 2 diabetes mellitus (T2D) and obesity (see Supplementary Table 3c).

We attempted to select informative genes based on the *p*-value heuristics and contextuality using CHAT analysis for identifying high dimensional gene expression data for validation. The existing approach based on identifying contextually important neighbors is in accordance with the principle of scale free property of networks. In this study, many key genes along with their enriched pathways underlying molecular mechanisms of obesity, lupus, adipose tissue, fatty acid pathways were obtained (Figure 5). When the phenotypic traits were combined with the DEGs, we identified enriched pathways in the form of Wnt signaling, cadherin, growth hormone (GH), and p53 pathways (Supplementary Table 2). These pathways were filtered based on the number of contextual neighbors the causal genes have. For example, the GH is well-known for understanding lean body mass, metabolism of muscle and bones while reducing body fat. From the concordant list, we observed that the genes related to cell growth, differentiation, apoptosis, immune function, brain function, and aging were enriched. With the GH also having insulin-like effects, it is expected that the genes related to stimulating amino acid transport, protein synthesis, glucose transport and lipogenesis form the same group. From the six phenotypic traits associated with the hubs, the GH receptor appears to be associated with tyrosine kinase JAK2. This indicates that they recruit signaling molecules such as STAT5 and Src family kinases and the availability of GHR on the cell surface is thus regulated.

On the other hand, activation of the p53 pathway in adipose tissues is mediated by insulin resistance in AIT2DM (Strycharz et al., 2017). Reports showed that higher level of DNA oxidation and a reduction in telomere length in adipose tissue would increase in DNA damage and activation of the p53 pathway in adipocytes (Vergoni et al., 2016). While various *in vivo* and *in vitro* studies have confirmed its role as inhibitor of white adipogenesis, an alleviate diet induced obesity is observed further exerting a positive effect on brown adipocyte differentiation program contributing to energy homeostasis in tumor suppression (Molchadsky et al., 2013). Our study reveals that DNA damage responsive pathways causal to obese adipocytes might trigger p53-dependent signals reflecting adipose tissue inflammation and dysfunction leading to insulin resistance in these subjects. So, p53 mediated obesity is not ruled out especially keeping in view of the HOMA-IR and HOMA-ß regulated pathways (Figure 3). The contextual nodes are shown in green while the non-contextual nodes are gray in color and circled. On the other hand, a good number of protein-coupled receptors (GPCRs) are expressed in our subjects where the M(3) muscarinic acetylcholine (ACh) receptor (M(3) mAChR) pathway preferentially is involved in peripheral tissues indicating that pancreatic ß-cells producing mAChRs are involved in glucose homeostasis and insulin sensitivity. This also complements our study result that there is a potential interest for the sick thigh fat mediated obesity underlining the metabolic disorders (Gautam et al., 2006). The GPCRs as discussed above are known to regulate the metabolic syndromes (MetS) and AIT2DM associated ailments which in turn serve as receptor antagonists. Reports on adiposity and defective insulin-mediated vasodilation have shown that the substrates are delivered to peripheral tissues (Harmelen et al., 2008). The distinct association of these hubs in our study show considerable evidence for understanding the pathophysiology of adiposity-related cell dysfunction. In addition, enrichment of mAChRs 1 and 3 signaling pathway has also been reported to associate with energy homeostasis against obesity-associated glucose intolerance, insulin resistance, hyperinsulinemia, and hyperglycemia conferring it a potential pharmacologic target for the treatment of obesity and associated metabolic disorders (Gautam et al., 2006). Another well-documented pathway is the corticotropin-releasing factor receptor type-2 (CRFR2). Expressed in ventromedial nucleus of hypothalamus (VMH) of the brain, it accumulates more white adipose tissue (WAT) than wild type (Chao et al., 2012) and their presence in adipose tissue has also been characterized (Seres et al., 2004) indicating that corticotropin-releasing hormone (CRH) system could be implicated in energy homeostasis and in mediating the anorexic effects of CRH at adipose level.

### Role of Wnt Signaling and Leptin Mediated Gonadotropin Releasing Hormones

Connecting adiponectin-related and dysfunction in fatty acid pathways and their key role in regulation is well-known (Xu et al., 2015). In our analyses, we observed the phenome-interactome pairs to be highly involved with Wnt and Cadherin signaling pathways (400 nodes each). This is in agreement with the fact that AIT2DM subjects' high fat diet might have an increased account of intestinal tumorigenesis through which the carriers of these variants have a profound significance toward regulation of this signaling pathway (Bordonaro, 2009). It may be argued that the proglucagon expression could be variable in these subjects that may explain the association of Wnt signaling with AIT2DM. The glucagon-like peptide-1 (GLP-1) and cadherin related ß-cell protection is a potential treatment for AIT2DM subjects. While we categorized the neighboring genes of enriched DEGs that fall into distinctly AIT2DM associated, interestingly the numbers of expression neighbors were observed to be significantly several times higher than for the rest of the genes (Figures 2, 3). Though the genes in these loci have little known impact in obesity, we identified many more known AIT2DM related important genes with a higher degree of connection further validating our approach. Our work supports that the AIT2DM contextual attributes signify the phenome-interactome relation which is in accordance with the enriched pathways, *viz*. Wnt signaling, Cadherin signaling, Heterotrimeric G-protein signaling, Gs alpha mediated, and Nicotinic acetylcholine receptor signaling pathways. In addition, the role of transforming growth factor β (TGFβ) as negative regulator of insulin signaling has been well-documented where TGFβ exerts its effect by inhibiting adipose cell differentiation by stimulating the expression and incorporation of fibronectin and collagen into the extra cellular matrix (ECM). This increase in ECM synthesis has been demonstrated to inhibit insulin signaling in 3T3-L1: a commonly used model of adipose cell differentiation (Gagnon et al., 1998). Furthermore, high expression of Wnt5a in visceral adipose tissue when compared with subcutaneous adipose tissue in obese individuals is known to enhance the expression of proinflammatory cytokines by macrophages in a Jun NH2-terminal kinase–dependent manner, leading to defective insulin signaling in adipocytes; Wnt5a-deficient knockoutmice (Wnt5a-KO) have been found to ameliorate high glucose and insulin level than wild type mice (Fuster et al., 2015).

Leptin, an adipose-hormone has been reported to stimulate release of gonadotropin-releasing hormone (GnRH) as part of its normal physiological role on sexual maturation (Attoub et al., 1999; Sanchez-Garrido and Tena- Sempere, 2013) increasing amount of its due to leptin-resistance in AIT2DM subjects may be accounted to enrichment of this pathway in our study. A positive regulator of phospholipase C-γ (PLCγ), GnRH however may have potential to attenuate insulin signaling due to hydrolysis by PLCγ of phosphatidylinositol 4, 5-bisphosphate (PIP2) into diacylglycerol (DAG) and inositol triphosphate (IP3) leading to insulin resistance (Saxena et al., 2017). Another pathway which regulates downstream nuclear factor κB, interferon regulatory factors, and STAT-1 activation (Kim et al., 2012) contributing to inflammation is toll-like receptors (TLR). Additionally, binge eating leading to adipogenic traits and disturbances in endogenous opioid systems (Giuliano and Cottone, 2015) and the whole lot of pathway associated genes augment the nutritional overflow hypothesis.

### Sick Thigh Fat as a Measure to Correlate Distinct Hubs

Our findings beyond doubt strongly support the concept of “sick fat or adiposopathy,” specifically “sick thigh fat” as one of the underlying diseases (i.e., organopathy) of AIT2DM (Bays, 2005). In light of emerging therapies like life style intervention (including Indian systems of yoga, traditional Indian diet, spices and herbs etc.) and medicines currently in practice (like saroglitazar, pioglitazone, salsalate) or in pipeline, which primarily intervene at level of treating “sick fat or adiposopathy,” the findings of this study not only provide strong evidences toward its molecular pathology, but could also be the corner stone for the future development of its diagnostic criteria. As adipose tissue dysfuncion and insulin resistance precedes ß cell dysfunction in the natural history of diabetes, strategy for pre-primary prevention of diabetes at the stage of adiposopathy alone, even before any glycemic dysfunction is expected to be much more effective strategy than the current strategy of primary prevention at the stage of impaired glucose tolerance (IGT), where the ß cell dysfunction already sets in. Given this state-of-the-art, we expected a good number of non-coding genes from our list of DEGs which could serve as diagnostic and prognostic biomarkers for these conditions. In addition, the role of intergenic/intragenic non-coding RNAs in systems integration of phenome interactomes for characterizing transcription signatures may be investigated. This is in agreement with the fact that the genetic variation underlying the pathogenesis of diseases can be best seen in non-coding regions of the genome. As one of the top enriched genes is a lncRNA (LINC01128) (Supplementary Table 3a), understanding functional studies with the non-coding genes revealing the distinct hubs portraying the phenotypic signatures cannot be ruled out. Furthermore, the negative correlation and the role of adipose tissue dysfunction could be associated with the pathways regulating the ß-cell function. The findings of this study have established beyond doubt that diabetes is associated with a molecular pathologic changes in thigh adipose tissue or vice versa. With the database of molecular signatures of various adipose tissues in terms of genome-wide gene expression profile made available in public domain for further bio-medical research (http://www.type2diabetesgenetics.org), analyzing genetic information from a wide array of phenome-interactome levels is likely possible. While we observed marked differences in clinical, biochemical, and radiological, cellular and molecular phenotypes, the role of qualitative changes in the form of phenotypic traits associated with the DEGs and the hubs positively correlating with the onset of diabetes is established.

## Conclusions

Identification of distinct hubs containing the DEGs would serve as circulatory biomarkers of adipose tissue pathology. From our analyses, the thigh adipose tisssue in AIT2DM subjects showed pathologic alteration, the so called “sick thigh fat” characterized by a large number of DEGs enriching distinct transriptional signatures. These transcription signatures were further characterizing using the phenome-interactome maps, where each of the traits under study including the intermediary phenotypes were found to be associated with distinct set of genes forming the hubs. These hubs, containing the DEGs (including non-coding RNAs associated with the novel pathways) can serve as biomarkers of thigh adipose sick fat for understanding the pathogenesis of AIT2DM. Additionally, they are also known to play a role in the process of adipogenesis, and therefore support the nutrient overflow hypothesis of Asian Indian phenotype. Notable among them are LINC01128 enriched with regulatory elements, RAS Guanyl Nucleotide-releasing Protein (RASGRP1) associated with high fat diet induced obesity enhancing adipocytes and lymphaniogenesis which could be plausible drivers for unhealthy fat depositions inside the body. In addition, we have the chance to study markers for healthy fat, where researchers have identified the adipose tissue specific fat markers in animal genomes (eutherians such as pig, cattle etc.) but as this study is contemplating the human subjects of interest, we haven't mapped the phenome interactome networks with eutherian population albeit the fact that LINC01128, RASGRP1, and PPARG which we have shown could be diet based markers. As we have identified various clinical, biochemical and radiological parameters, which show significant correlation with these hubs, therefore, these clinical parameters can possibly be taken as surrogate markers of sick thigh fat for epidemiological study purpose and the creation of criteria for the diagnosis of various stages of this organopathy. There is need of further investigation, comparing the process of adipogenesis and sick thigh fat in different depots of adipose tissue, including larger number of subjects, considering gender dichotomy and across the various stages of natural history of diabetes.

## Author Contributions

PT and SM were involved in collecting samples, biochemical characterization of the tissues. PT performed the microarray analyses of the samples. AS and PS analyzed the DEGs using RMA. PT, NG, and KM performed qPCR validation. PT and PS wrote the first draft. PS developed the systems integration of phenotypic traits. SM, KM, and PS proofread the manuscript. All authors read and approved the proof of the manuscript before submission.

### Conflict of Interest Statement

The authors declare that the research was conducted in the absence of any commercial or financial relationships that could be construed as a potential conflict of interest.
