# Supplementary material for: Systems Genomics of Thigh Adipose Tissue From Asian Indian Type-2 Diabetics Revealed Distinct Protein Interaction Hubs
Source: Front Genet. 2019 Jan 8;9:679. doi: 10.3389/fgene.2018.00679 (PMC6331691; doi:10.3389/fgene.2018.00679)
Supplement: Supplementary file 4 [file Table_4.doc]

**Supplementary table 4a: Pipeline for RMA analyses forThree prime Affymetrix Arrays :**

# load the affy library
library(affy)

# Reading the CEL files from prash/projects/limbfat/CEL/

data <- ReadAffy() 
eset <- rma(data)

# log2 transform and normalization
write.exprs(eset,file="data.txt")

### Adding Gene Annotation to Normalized Expression Output

library(hugene10sttranscriptcluster.db)

# Putting expression info into a data frame
my_frame <- data.frame(exprs(eset))

# Put annotation

Annot <- data.frame(ACCNUM=sapply(contents(hugene10sttranscriptclusterACCNUM), paste, collapse=", "), SYMBOL=sapply(contents(hugene10sttranscriptclusterSYMBOL), paste, collapse=", "), DESC=sapply(contents(hugene10sttranscriptclusterGENENAME), paste, collapse=", "))

# Merging data frames
all <- merge(Annot, my_frame, by.x=0, by.y=0, all=T)

# Write out to a file:
write.table(all,file="data.ann.txt",sep="\t")

**Supplementary table 4b: Pipeline for cDNA synthesis and hybridization**

RNA isolated from Adipose tissue and quantified.

RNA sample prepared with Poly-A RNA Controls mix

First-Strand and second-strand cDNA synthesized

Labeled cRNA synthesized by in vitro transcription by addition of Biotinylated Ribonucleotide

Labeled cRNA purified by using bead based purification method

Labeled cRNA Yield assessed using Nanodrop/ spectrophotometer

Labeled cRNA fragmented using fragmentation buffer

The fragmented labelled cRNA hybridised on GeneChip PrimeView Human Gene Expression Array Array

The arrays hybridised in GeneChip Hybridization oven 645

After hybridization, the arrays washed and stained on Affymmetrix Gene Chip Fluidic station 450 Dx and scanned on GeneChip Scanner

Raw Data generated and quality check (QC) of the data is then checked using Affymetrix expression console and Transcriptomic Analysis suite 4.0 software
