# Supplementary material for: Systems Genomics of Thigh Adipose Tissue From Asian Indian Type-2 Diabetics Revealed Distinct Protein Interaction Hubs
Source: Front Genet. 2019 Jan 8;9:679. doi: 10.3389/fgene.2018.00679 (PMC6331691; doi:10.3389/fgene.2018.00679)
Supplement: Supplementary file 5 [file Table_5.docx]

Supplementary Table 5: Selected genes for quantitative RT-PCR

|  | **NON DIABETIC** | **Average** | **Std Dev** | **Cp(ND)-Cp(C)** |  |  |
| --- | --- | --- | --- | --- | --- | --- |
| **S.No.** | **Gene Name** |  |  | **C1** |  |  |
| **1** | **ISG15** | **38.56** | **3.13** | **7.21** |  |  |
| **2** | **CALML6** | **42.12** | **4.09** | **10.77** |  |  |
| **3** | **VWA1** | **32.66** | **1.71** | **1.31** |  |  |
| **4** | **SOS1** | **37.05** | **1.66** | **5.70** |  |  |
| **5** | **SLC33A1** | **38.97** | **7.55** | **7.62** |  |  |
| **6** | **MUSTN1** | **40.41** | **2.28** | **9.06** |  |  |
| **7** | **CTIF** | **32.67** | **4.53** | **1.32** |  |  |
| **8** | **ACTB** | **33.34** | **2.08** | **1.99** |  |  |
| **9** | **G6PD** | **31.35** | **3.68** | **0.00** |  |  |
|  |  |  |  |  |  |  |
|  |  |  |  |  |  |  |
|  | **DIABETIC** | **Average** | **Std Dev** | **Cp(DM)-Cp(C)** | **(C3-C1)** | **(C3-C1)X2** |
| **S.No.** | **Gene Name** |  |  | **C3** |  |  |
| **1** | **ISG15** | **36.58** | **1.31** | **6.21** | **-1.00** | **-1.99** |
| **2** | **CALML6** | **36.08** | **3.70** | **5.71** | **-5.06** | **-10.11** |
| **3** | **VWA1** | **32.77** | **1.66** | **2.40** | **1.09** | **2.18** |
| **4** | **SOS1** | **36.86** | **1.13** | **6.49** | **0.79** | **1.58** |
| **5** | **SLC33A1** | **38.32** | **4.58** | **7.95** | **0.33** | **0.66** |
| **6** | **MUSTN1** | **40.53** | **4.53** | **10.16** | **1.10** | **2.19** |
| **7** | **CTIF** | **31.06** | **2.06** | **0.69** | **-0.64** | **-1.27** |
| **8** | **ACTB** | **32.54** | **2.33** | **2.17** | **0.18** | **0.36** |
| **9** | **G6PD** | **30.37** | **1.40** | **0.00** | **0.00** | **0.00** |

| **Gene Name** | **NON DIABETIC** | **Std Dev** | **DIABETIC** | **Std Dev** |
| --- | --- | --- | --- | --- |
| **ISG15** | **38.56** | **3.13** | **36.58** | **1.31** |
| **CALML6** | **42.12** | **4.09** | **36.08** | **3.70** |
| **VWA1** | **32.66** | **1.71** | **32.77** | **1.66** |
| **SOS1** | **37.05** | **1.66** | **36.86** | **1.13** |
| **SLC33A1** | **38.97** | **7.55** | **38.32** | **4.58** |
| **MUSTN1** | **40.41** | **2.28** | **40.53** | **4.53** |
| **CTIF** | **32.67** | **4.53** | **31.06** | **2.06** |
| **ACTB** | **33.34** | **2.08** | **32.54** | **2.33** |
| **G6PD** | **31.35** | **3.68** | **30.37** | **1.40** |
